# 4-(4-Pentyl­cyclo­hexyl)phenol

**DOI:** 10.1107/S1600536809024027

**Published:** 2009-06-27

**Authors:** He-Fang Wang, Yong Guo, Chang-Qing Jin, Hong-Bing Le

**Affiliations:** a School of Chemical Engineering and Technology, Hebei University of Technology, Tianjin 300131, People’s Republic of China; b CNOOC Tianjin Research and Design Institute of Chemical Industry, Tianjin 300131, People’s Republic of China

## Abstract

In the title compound, C_17_H_26_O, the cyclo­hexyl ring adopts a chair conformation with the C-atom substituents in equatorial sites. The H atom of the O—H group is disordered over two positions of equal occupancy. In the crystal, O—H⋯O hydrogen bonds lead to [010] chains.

## Related literature

For a related structure, see: Wang *et al.* (2006 ▶). For applications of phenol derivatives, see: Eidenschink *et al.* (1978 ▶); Hu *et al.* (2003 ▶).
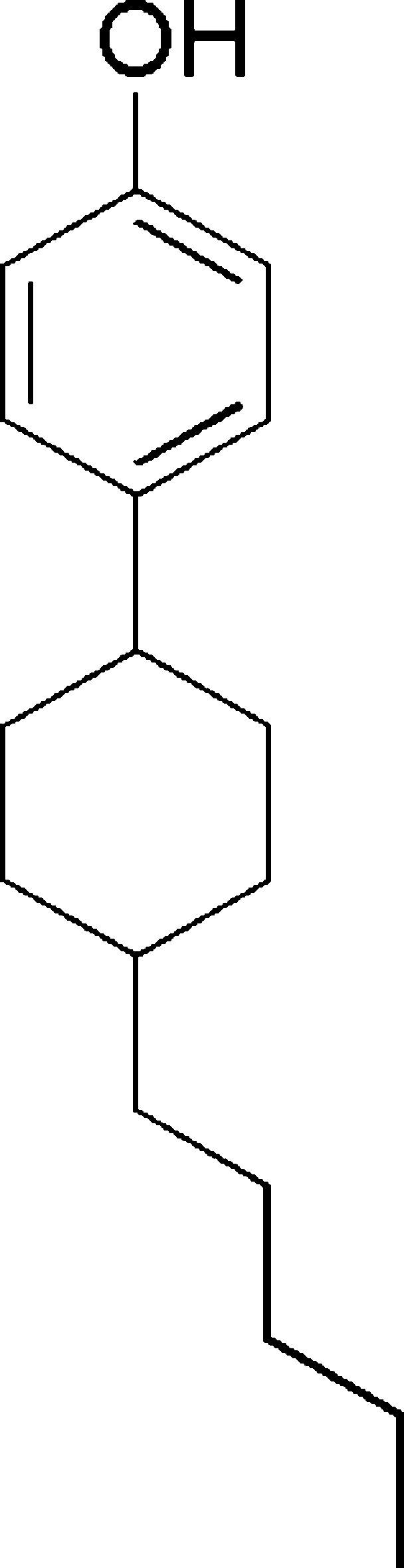


## Experimental

### 

#### Crystal data

C_17_H_26_O
*M* *_r_* = 246.38
Monoclinic, 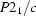

*a* = 21.002 (4) Å
*b* = 5.3281 (11) Å
*c* = 13.389 (3) Å
β = 105.87 (3)°
*V* = 1441.2 (5) Å^3^
*Z* = 4
Mo *K*α radiation
μ = 0.07 mm^−1^
*T* = 113 K
0.24 × 0.20 × 0.10 mm

#### Data collection

Rigaku Saturn CCD diffractometer
Absorption correction: multi-scan (*CrystalClear*; Rigaku/MSC, 2005 ▶) *T*_min_ = 0.984, *T*_max_ = 0.993
10687 measured reflections
2827 independent reflections
2314 reflections with *I* > 2σ(*I*)
*R*_int_ = 0.040

#### Refinement

*R*[*F* ^2^ > 2σ(*F* ^2^)] = 0.056
*wR*(*F* ^2^) = 0.151
*S* = 1.11
2827 reflections
170 parameters
2 restraints
H atoms treated by a mixture of independent and constrained refinement
Δρ_max_ = 0.19 e Å^−3^
Δρ_min_ = −0.25 e Å^−3^

### 

Data collection: *CrystalClear* (Rigaku/MSC, 2005 ▶); cell refinement: *CrystalClear*; data reduction: *CrystalClear*; program(s) used to solve structure: *SHELXS97* (Sheldrick, 2008 ▶); program(s) used to refine structure: *SHELXL97* (Sheldrick, 2008 ▶); molecular graphics: *SHELXTL* (Sheldrick, 2008 ▶); software used to prepare material for publication: *SHELXTL*.

## Supplementary Material

Crystal structure: contains datablocks I, global. DOI: 10.1107/S1600536809024027/hb5011sup1.cif

Structure factors: contains datablocks I. DOI: 10.1107/S1600536809024027/hb5011Isup2.hkl

Additional supplementary materials:  crystallographic information; 3D view; checkCIF report

## Figures and Tables

**Table 1:** Hydrogen-bond geometry (Å, °)

*D*—H⋯*A*	*D*—H	H⋯*A*	*D*⋯*A*	*D*—H⋯*A*
O1—H1*B*⋯O1^i^	0.84 (2)	2.06 (2)	2.886 (2)	170 (4)
O1—H1*A*⋯O1^ii^	0.87 (2)	1.99 (2)	2.836 (2)	165 (4)

Symmetry codes: (i) 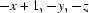; (ii) 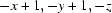.

**Fig. 1.**
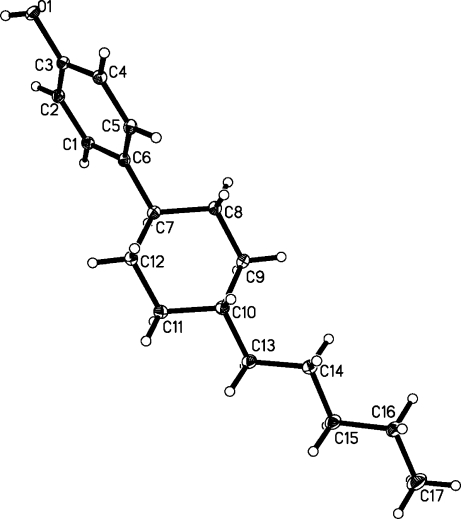
The molecular structure of (I). Displacement ellipsoids are drawn at the 30% probability level and H atoms are shown as small spheres of arbitrary radius. Only one orientation of the O—H group is shown.

## supplementary crystallographic information

### Comment

For a related structure, see Wang *et al.* (2006); for uses of phenol derivatives, see Eidenschink *et al.*, 1978; Hu *et al.*, 2003).  In the title compound, (I), the H atom of O—H bond was found disordered in two orientation. The crystal structure is stabilized by O—H···O hydrogen bonds (Table 1).

### Refinement

The H atoms of O—H were located in a difference map and their positions were freely refined. All H other atoms were positioned geometrically and refined using a riding model, in the range of 0.93–0.98 Å, with *U*_iso_(H) = 1.2*U*_eq_(C) or 1.5*U*_eq_(methyl C).

### Crystal data

**Table:**

C_17_H_26_O	*F*(000) = 544
*M**_r_* = 246.38	*D*_x_ = 1.136 Mg m^−^^3^
Monoclinic, *P*2_1_/*c*	Mo *K*α radiation, λ = 0.71073 Å
Hall symbol: -P 2ybc	Cell parameters from 3895 reflections
*a* = 21.002 (4) Å	θ = 1.6–27.9°
*b* = 5.3281 (11) Å	µ = 0.07 mm^−^^1^
*c* = 13.389 (3) Å	*T* = 113 K
β = 105.87 (3)°	Block, colourless
*V* = 1441.2 (5) Å^3^	0.24 × 0.20 × 0.10 mm
*Z* = 4	

### Data collection

**Table:**

Rigaku Saturn CCD diffractometer	2827 independent reflections
Radiation source: rotating anode	2314 reflections with *I* > 2σ(*I*)
confocal	*R*_int_ = 0.040
Detector resolution: 7.31 pixels mm^-1^	θ_max_ = 26.0°, θ_min_ = 2.0°
ω and φ scans	*h* = −25→22
Absorption correction: multi-scan (*CrystalClear*; Rigaku/MSC, 2005)	*k* = −6→6
*T*_min_ = 0.984, *T*_max_ = 0.993	*l* = −16→15
10687 measured reflections	

### Refinement

**Table:**

Refinement on *F*^2^	Primary atom site location: structure-invariant direct methods
Least-squares matrix: full	Secondary atom site location: difference Fourier map
*R*[*F*^2^ > 2σ(*F*^2^)] = 0.056	Hydrogen site location: inferred from neighbouring sites
*wR*(*F*^2^) = 0.151	H atoms treated by a mixture of independent and constrained refinement
*S* = 1.11	*w* = 1/[σ^2^(*F*_o_^2^) + (0.0738*P*)^2^ + 0.2552*P*] where *P* = (*F*_o_^2^ + 2*F*_c_^2^)/3
2827 reflections	(Δ/σ)_max_ = 0.001
170 parameters	Δρ_max_ = 0.19 e Å^−^^3^
2 restraints	Δρ_min_ = −0.25 e Å^−^^3^

### Special details

**Table:**

Geometry. All e.s.d.'s (except the e.s.d. in the dihedral angle between two l.s. planes) are estimated using the full covariance matrix. The cell e.s.d.'s are taken into account individually in the estimation of e.s.d.'s in distances, angles and torsion angles; correlations between e.s.d.'s in cell parameters are only used when they are defined by crystal symmetry. An approximate (isotropic) treatment of cell e.s.d.'s is used for estimating e.s.d.'s involving l.s. planes.
Refinement. Refinement of *F*^2^ against ALL reflections. The weighted *R*-factor *wR* and goodness of fit *S* are based on *F*^2^, conventional *R*-factors *R* are based on *F*, with *F* set to zero for negative *F*^2^. The threshold expression of *F*^2^ > σ(*F*^2^) is used only for calculating *R*-factors(gt) *etc*. and is not relevant to the choice of reflections for refinement. *R*-factors based on *F*^2^ are statistically about twice as large as those based on *F*, and *R*- factors based on ALL data will be even larger.

### Fractional atomic coordinates and isotropic or equivalent isotropic displacement parameters (Å^2^)

**Table:**

	*x*	*y*	*z*	*U*_iso_*/*U*_eq_	Occ. (<1)
O1	0.48580 (6)	0.2525 (2)	0.02646 (9)	0.0245 (3)	
H1A	0.4889 (19)	0.399 (5)	−0.001 (3)	0.037*	0.50
H1B	0.4982 (19)	0.107 (5)	0.018 (3)	0.037*	0.50
C1	0.41419 (7)	0.0832 (3)	0.23796 (11)	0.0186 (3)	
H1	0.4178	−0.0466	0.2878	0.022*	
C2	0.45317 (7)	0.0725 (3)	0.16908 (11)	0.0195 (3)	
H2	0.4833	−0.0621	0.1723	0.023*	
C3	0.44742 (7)	0.2600 (3)	0.09596 (11)	0.0189 (3)	
C4	0.40401 (7)	0.4582 (3)	0.09173 (11)	0.0199 (4)	
H4	0.4006	0.5876	0.0417	0.024*	
C5	0.36564 (7)	0.4663 (3)	0.16100 (11)	0.0197 (4)	
H5	0.3357	0.6018	0.1576	0.024*	
C6	0.36995 (7)	0.2801 (3)	0.23555 (11)	0.0180 (3)	
C7	0.32602 (7)	0.2816 (3)	0.30842 (11)	0.0183 (4)	
H7	0.3429	0.1491	0.3621	0.022*	
C8	0.32710 (7)	0.5321 (3)	0.36579 (11)	0.0205 (4)	
H8A	0.3729	0.5679	0.4074	0.025*	
H8B	0.3132	0.6685	0.3142	0.025*	
C9	0.28151 (7)	0.5298 (3)	0.43741 (11)	0.0211 (4)	
H9A	0.2820	0.6977	0.4694	0.025*	
H9B	0.2985	0.4070	0.4939	0.025*	
C10	0.20997 (7)	0.4612 (3)	0.37901 (11)	0.0196 (4)	
H10	0.1929	0.5933	0.3253	0.024*	
C11	0.20944 (8)	0.2107 (3)	0.32292 (12)	0.0215 (4)	
H11A	0.2240	0.0759	0.3751	0.026*	
H11B	0.1636	0.1722	0.2820	0.026*	
C12	0.25437 (7)	0.2132 (3)	0.25071 (12)	0.0208 (4)	
H12A	0.2372	0.3362	0.1944	0.025*	
H12B	0.2536	0.0455	0.2186	0.025*	
C13	0.16400 (8)	0.4506 (3)	0.44966 (12)	0.0227 (4)	
H13A	0.1214	0.3758	0.4101	0.027*	
H13B	0.1838	0.3369	0.5084	0.027*	
C14	0.14968 (8)	0.7019 (3)	0.49357 (12)	0.0238 (4)	
H14A	0.1917	0.7737	0.5366	0.029*	
H14B	0.1315	0.8191	0.4354	0.029*	
C15	0.10106 (8)	0.6811 (3)	0.55919 (12)	0.0258 (4)	
H15A	0.0596	0.6038	0.5167	0.031*	
H15B	0.1200	0.5676	0.6184	0.031*	
C16	0.08453 (8)	0.9309 (3)	0.60091 (13)	0.0273 (4)	
H16A	0.1262	1.0150	0.6386	0.033*	
H16B	0.0621	1.0395	0.5417	0.033*	
C17	0.04046 (9)	0.9051 (4)	0.67340 (14)	0.0402 (5)	
H17A	−0.0006	0.8194	0.6371	0.060*	
H17B	0.0301	1.0721	0.6953	0.060*	
H17C	0.0635	0.8071	0.7345	0.060*	

### Atomic displacement parameters (Å^2^)

**Table:**

	*U*^11^	*U*^22^	*U*^33^	*U*^12^	*U*^13^	*U*^23^
O1	0.0299 (7)	0.0234 (6)	0.0256 (7)	0.0001 (5)	0.0169 (5)	−0.0007 (5)
C1	0.0216 (8)	0.0165 (7)	0.0183 (8)	−0.0007 (6)	0.0064 (6)	0.0006 (6)
C2	0.0201 (7)	0.0172 (7)	0.0217 (8)	0.0017 (6)	0.0065 (6)	−0.0008 (6)
C3	0.0194 (8)	0.0214 (8)	0.0168 (8)	−0.0025 (6)	0.0066 (6)	−0.0024 (6)
C4	0.0239 (8)	0.0181 (7)	0.0172 (8)	−0.0014 (6)	0.0051 (6)	0.0009 (6)
C5	0.0223 (8)	0.0174 (7)	0.0196 (8)	0.0027 (6)	0.0059 (6)	−0.0005 (6)
C6	0.0194 (8)	0.0189 (8)	0.0152 (8)	−0.0010 (6)	0.0038 (6)	−0.0020 (6)
C7	0.0204 (8)	0.0183 (8)	0.0170 (8)	0.0013 (6)	0.0063 (6)	0.0015 (6)
C8	0.0206 (8)	0.0209 (8)	0.0205 (8)	−0.0013 (6)	0.0066 (6)	−0.0026 (6)
C9	0.0226 (8)	0.0220 (8)	0.0200 (8)	0.0013 (6)	0.0083 (7)	−0.0022 (6)
C10	0.0223 (8)	0.0192 (8)	0.0183 (8)	0.0000 (6)	0.0074 (6)	0.0010 (6)
C11	0.0222 (8)	0.0217 (8)	0.0218 (8)	−0.0030 (6)	0.0081 (7)	−0.0015 (6)
C12	0.0237 (8)	0.0189 (8)	0.0210 (8)	−0.0009 (6)	0.0079 (7)	−0.0017 (6)
C13	0.0246 (8)	0.0229 (8)	0.0230 (8)	−0.0007 (6)	0.0105 (7)	0.0006 (6)
C14	0.0248 (8)	0.0254 (9)	0.0240 (9)	−0.0008 (6)	0.0112 (7)	−0.0006 (6)
C15	0.0282 (9)	0.0278 (9)	0.0249 (9)	0.0001 (7)	0.0134 (7)	0.0007 (7)
C16	0.0257 (9)	0.0326 (10)	0.0260 (9)	−0.0005 (7)	0.0112 (7)	−0.0052 (7)
C17	0.0337 (10)	0.0584 (13)	0.0346 (11)	−0.0006 (9)	0.0193 (9)	−0.0108 (9)

### Geometric parameters (Å, °)

**Table:**

O1—C3	1.3883 (17)	C10—C13	1.5264 (19)
O1—H1A	0.87 (2)	C10—C11	1.530 (2)
O1—H1B	0.84 (2)	C10—H10	1.0000
C1—C2	1.3918 (19)	C11—C12	1.5245 (19)
C1—C6	1.396 (2)	C11—H11A	0.9900
C1—H1	0.9500	C11—H11B	0.9900
C2—C3	1.381 (2)	C12—H12A	0.9900
C2—H2	0.9500	C12—H12B	0.9900
C3—C4	1.386 (2)	C13—C14	1.525 (2)
C4—C5	1.3856 (19)	C13—H13A	0.9900
C4—H4	0.9500	C13—H13B	0.9900
C5—C6	1.393 (2)	C14—C15	1.5222 (19)
C5—H5	0.9500	C14—H14A	0.9900
C6—C7	1.5151 (19)	C14—H14B	0.9900
C7—C12	1.536 (2)	C15—C16	1.520 (2)
C7—C8	1.5373 (19)	C15—H15A	0.9900
C7—H7	1.0000	C15—H15B	0.9900
C8—C9	1.5291 (19)	C16—C17	1.520 (2)
C8—H8A	0.9900	C16—H16A	0.9900
C8—H8B	0.9900	C16—H16B	0.9900
C9—C10	1.536 (2)	C17—H17A	0.9800
C9—H9A	0.9900	C17—H17B	0.9800
C9—H9B	0.9900	C17—H17C	0.9800
			
C3—O1—H1A	112 (3)	C9—C10—H10	107.9
C3—O1—H1B	112 (3)	C12—C11—C10	112.39 (12)
H1A—O1—H1B	135 (4)	C12—C11—H11A	109.1
C2—C1—C6	121.60 (13)	C10—C11—H11A	109.1
C2—C1—H1	119.2	C12—C11—H11B	109.1
C6—C1—H1	119.2	C10—C11—H11B	109.1
C3—C2—C1	119.14 (13)	H11A—C11—H11B	107.9
C3—C2—H2	120.4	C11—C12—C7	111.98 (12)
C1—C2—H2	120.4	C11—C12—H12A	109.2
C2—C3—C4	120.62 (14)	C7—C12—H12A	109.2
C2—C3—O1	120.02 (13)	C11—C12—H12B	109.2
C4—C3—O1	119.36 (13)	C7—C12—H12B	109.2
C5—C4—C3	119.51 (13)	H12A—C12—H12B	107.9
C5—C4—H4	120.2	C14—C13—C10	115.56 (12)
C3—C4—H4	120.2	C14—C13—H13A	108.4
C4—C5—C6	121.46 (13)	C10—C13—H13A	108.4
C4—C5—H5	119.3	C14—C13—H13B	108.4
C6—C5—H5	119.3	C10—C13—H13B	108.4
C5—C6—C1	117.67 (13)	H13A—C13—H13B	107.5
C5—C6—C7	121.73 (13)	C15—C14—C13	113.12 (13)
C1—C6—C7	120.52 (13)	C15—C14—H14A	109.0
C6—C7—C12	111.11 (11)	C13—C14—H14A	109.0
C6—C7—C8	113.45 (12)	C15—C14—H14B	109.0
C12—C7—C8	109.63 (12)	C13—C14—H14B	109.0
C6—C7—H7	107.5	H14A—C14—H14B	107.8
C12—C7—H7	107.5	C16—C15—C14	113.88 (13)
C8—C7—H7	107.5	C16—C15—H15A	108.8
C9—C8—C7	112.35 (12)	C14—C15—H15A	108.8
C9—C8—H8A	109.1	C16—C15—H15B	108.8
C7—C8—H8A	109.1	C14—C15—H15B	108.8
C9—C8—H8B	109.1	H15A—C15—H15B	107.7
C7—C8—H8B	109.1	C17—C16—C15	113.29 (14)
H8A—C8—H8B	107.9	C17—C16—H16A	108.9
C8—C9—C10	112.06 (12)	C15—C16—H16A	108.9
C8—C9—H9A	109.2	C17—C16—H16B	108.9
C10—C9—H9A	109.2	C15—C16—H16B	108.9
C8—C9—H9B	109.2	H16A—C16—H16B	107.7
C10—C9—H9B	109.2	C16—C17—H17A	109.5
H9A—C9—H9B	107.9	C16—C17—H17B	109.5
C13—C10—C11	110.54 (12)	H17A—C17—H17B	109.5
C13—C10—C9	112.94 (12)	C16—C17—H17C	109.5
C11—C10—C9	109.41 (12)	H17A—C17—H17C	109.5
C13—C10—H10	107.9	H17B—C17—H17C	109.5
C11—C10—H10	107.9		
			
C6—C1—C2—C3	−0.6 (2)	C12—C7—C8—C9	54.21 (15)
C1—C2—C3—C4	0.8 (2)	C7—C8—C9—C10	−55.74 (16)
C1—C2—C3—O1	−179.92 (12)	C8—C9—C10—C13	178.39 (12)
C2—C3—C4—C5	−0.7 (2)	C8—C9—C10—C11	54.80 (16)
O1—C3—C4—C5	−179.99 (12)	C13—C10—C11—C12	179.57 (12)
C3—C4—C5—C6	0.4 (2)	C9—C10—C11—C12	−55.45 (16)
C4—C5—C6—C1	−0.3 (2)	C10—C11—C12—C7	56.67 (16)
C4—C5—C6—C7	−177.26 (13)	C6—C7—C12—C11	179.35 (11)
C2—C1—C6—C5	0.4 (2)	C8—C7—C12—C11	−54.44 (15)
C2—C1—C6—C7	177.41 (13)	C11—C10—C13—C14	−168.57 (13)
C5—C6—C7—C12	72.10 (17)	C9—C10—C13—C14	68.48 (17)
C1—C6—C7—C12	−104.78 (16)	C10—C13—C14—C15	177.28 (12)
C5—C6—C7—C8	−51.96 (18)	C13—C14—C15—C16	−178.21 (14)
C1—C6—C7—C8	131.16 (14)	C14—C15—C16—C17	−175.27 (14)
C6—C7—C8—C9	179.08 (12)		

### Hydrogen-bond geometry (Å, °)

**Table:**

*D*—H···*A*	*D*—H	H···*A*	*D*···*A*	*D*—H···*A*
O1—H1B···O1^i^	0.84 (2)	2.06 (2)	2.886 (2)	170 (4)
O1—H1A···O1^ii^	0.87 (2)	1.99 (2)	2.836 (2)	165 (4)

Symmetry codes: (i) −*x*+1, −*y*, −*z*; (ii) −*x*+1, −*y*+1, −*z*.
